# Alteration of Growth Performance, Antioxidant Capacity, Tissue Fatty Acid Profiles, and Lipid Metabolism of Mud Crab (*Scylla paramamosain*) Juvenile in Response to Different Dietary Arachidonic Acid Levels

**DOI:** 10.1155/2022/6038613

**Published:** 2022-11-23

**Authors:** Fang Fang, Ye Yuan, Min Jin, Yingying Zhang, Tingting Zhu, Jiaxiang Luo, Zheng Yang, Chen Guo, Lefei Jiao, Xiaojun Yan, Qicun Zhou

**Affiliations:** ^1^Laboratory of Fish and Shellfish Nutrition, School of Marine Sciences, Ningbo University, Ningbo 315211, China; ^2^Key Laboratory of Aquaculture Biotechnology Ministry of Education, Ningbo University, Ningbo 315211, China; ^3^Guangdong Provincial Key Laboratory of Marine Biotechnology, Institute of Marine Sciences, Shantou University, Shantou 515063, China

## Abstract

An eight-week feeding trail was carried out to investigate the impacts of different dietary arachidonic acid (ARA) supplementations on growth performance, antioxidant capacity, tissue fatty acid profiles, and lipid metabolism of mud crab (*Scylla paramamosain*) juvenile. Six isonitrogenous (480 g kg^−1^ crude protein) and isolipidic (80 g kg^−1^ crude lipid) diets were formulated to contain 0.40, 2.50, 4.60, 8.90, 12.50, and 15.70 g ARA kg^−1^ (dry matter), respectively. Each experimental treatment included 24 mud crab juveniles (initial weight 11.29 ± 0.09 g) and was assigned to triplicate groups (*n* = 3). Crabs fed diets with 2.50, 4.60, and 8.90 g kg^−1^ ARA presented significantly higher percent weight gain (PWG) and specific growth rate (SGR) than those fed the other diets. Based on two-slope broken-line and quadratic curve regression analysis of PWG against dietary ARA levels, optimal dietary ARA levels were determined to be 5.20 g kg^−1^ and 6.20 g kg^−1^, respectively. Crabs fed with 4.60 g kg^−1^ ARA diet showed the lowest activities of alanine aminotransferase (ALT) as well as aspartate aminotransferase (AST) in hemolymph among all treatments. In hemolymph and hepatopancreas, total antioxidant capacity (T-AOC), the activities of total superoxide dismutase (T-SOD), and glutathione peroxidase (GSH-Px) as well as the contents of reduced glutathione (GSH) rose first and then dropped with the increase of dietary ARA levels, while the concentration of malondialdehyde (MDA) showed an opposite trend. Tissue fatty acid profiles reflected diets fatty acid compositions. The ARA contents in hepatopancreas and muscle significantly increased with the increase of dietary ARA levels. Furthermore, the areas of blasenzellen (B) cells and restzellen (R) cells were significantly downregulated with the increase of dietary ARA levels. Crabs fed with 0.40 g kg^−1^ ARA diet showed significantly higher gene expression levels of fatty acid synthase (fas) as well as acetyl-CoA carboxylase (acc) among all treatments. Relative gene expression levels of 6-phosphogluconate dehydrogenase (6pgd) as well as glucose-6-phosphate dehydrogenase (g6pd) have been significantly upregulated in 0.40 and 2.50 g kg^−1^ ARA groups. Relative gene expression level of fatty acid binding protein 1 (fabp1) significantly increased in 4.60, 8.90, 12.50, and 15.70 g kg^−1^ ARA groups. However, the gene expression levels of fatty acid binding protein 4 (fabp4) as well as scavenger receptor class 2 (srb2) have not been influenced by dietary ARA levels. What is more, crabs fed diets with 4.60, 8.90, 12.50, and 15.70 g kg^−1^ ARA had a significantly higher expression level of carnitine palmitoyltransferase 1 (cpt1) than those fed diets with 0.40 and 2.50 g kg^−1^ ARA. In summary, optimum dietary ARA can promote growth, enhance antioxidant capacity, and improve health of mud crab juveniles. It also demonstrated that lipogenesis has been restrained with the increasing dietary ARA levels. These findings could provide theoretical guidance and reference for the lipid nutrition research as well as the development of the commercial diet in mud crab.

## 1. Introduction

In animal nutrition, lipids are crucial, served as major metabolic energy sources, membrane phospholipids components, and precursors of bioactive metabolites [1]. Long-chain polyunsaturated fatty acids (LC-PUFAs) contain 18-20 carbons or more, playing key roles in growth and development of aquatic animals. According to position of the first double bond from methyl end group, LC-PUFAs can be grossly divided into two groups (n-3 and n-6) [2]. In the past decades, numerous nutritional studies related to n-3 LC-PUFAs, particularly docosahexaenoic acid (DHA) and (eicosapentaenoic acid) EPA, have been reported in aquatic animals [3–7]. Significances of n-6 LC-PUFA, especially ARA, have been relatively overlooked as that ARA was generally required in a small amount [8]. As precursors of eicosanoids, including prostaglandins (PGs), thromboxanes, and leukotrienes (LTs), ARA has been reported to participate in regulating growth, reproduction, antioxidant capacity, immunity, fatty acid metabolism, etc. [9–13]. Usually, fishes can use the substrate of C18 polyunsaturated fatty acid to biosynthesis ARA [14]. But in crustaceans, they cannot synthesize ARA in adequate amounts as relative lack of Δ5 desaturase or Δ6 elongase [15]. Thus, ARA is an essential fatty acid for crustaceans and they must take it from diets to meet their needs. Recently, a number of studies have demonstrated that dietary ARA could influence growth, reproductive performances, immunity, and lipid metabolism in several crustacean species. In terms of shrimp species, dietary ARA promoted growth and improved immunity of oriental river prawn *Macrobrachium nipponense* [16]. And in Pacific white shrimp *Litopenaeus vannamei*, dietary ARA supplementations have improved final reproductive performances of at the early maturation stages and influenced relative expression levels of lipid-relevant genes [17]. Coman et al. also suggested that dietary ARA could significantly increase the spawning rate, spawning, and egg number of female shrimp, and thus played key roles in promoting egg development and spawning of black tiger prawn *Penaeus monodon* [18]. Although the mechanisms of dietary ARA functions in fish and shrimp have been studied, little information is available on the nutritional role of ARA in crabs, so far, only Chinese mitten crab (*Eriocheir sinensis*) has been determined that dietary ARA significantly affected its growth, feed utilization, fatty acid metabolism, immunity, and antioxidant capacities [19, 20].

Mud crab, *Scylla paramamosain*, is naturally distributed in Indo-Pacific coast [21]. It is an economically important crab species due to its high market value. In 2021, the mariculture production of mud crab was up to 152,065 tons, which occupied the first position among marine commercial crabs in China [22]. To date, rare studies related to the influences of ARA in mud crab have been reported. Notably, a recent study on postlarval mud crab *Scylla paramamosain* (initial weight 8.15 mg) indicated that moderate ARA level (11.80 g ARA kg^−1^ diet) improved growth, antioxidant capacity, and regulated lipid metabolism [23]. Differently, the present research is aimed at investigating impacts of ARA levels in diets on growth performance, antioxidant capacity, and tissue fatty acid profiles as well as lipid metabolism of mud crab juvenile (initial weight 11.29 ± 0.09 g). That will be beneficial to investigate the ARA requirements of mud crab at different growth stages.

## 2. Materials and Methods

### 2.1. Ethics Statement

Trial procedures were followed by Standard Operation Procedures (SOPs) in Experimental Animals Protocols (Ningbo University). This research has been also permitted by Scientific Ethics Committee for Experimental Animals (Ningbo University).

### 2.2. Experimental Diets

Six isonitrogenous (480 g protein kg^−1^ diet) and isolipidic (80 g lipid kg^−1^ diet) diets were produced with graded ARA levels (Table 1). Peru fish meal and soybean protein concentrate served as major protein sources. ARA-enriched oil, palmitic acid, and soybean lecithin were major lipid sources. Wheat flour served as main carbohydrate sources. ARA-enriched oil (500 g kg^−1^ content) was added at the expense of palmitin to obtain six groups, 0.40 g ARA kg^−1^ diet, 2.50 g ARA kg^−1^ diet, 4.60 g ARA kg^−1^ diet, 8.90 g ARA kg^−1^ diet, 12.50 g ARA kg^−1^ diet, and 15.70 g ARA kg^−1^ diet (measured values, dry matter). Diet fatty acid compositions are presented in Table 2. Methods of diet preparation were followed by the procedure described in detail previously [6]. In brief, fine powders of ingredients were blended in a Hobart type mixer, and then pellets were obtained by a F-26 twin screws extruder (Machine factory of South China University of Technology, Guangzhou, China). Pellets strands were cut into small size (diameter: 2.0 mm, length: 3.0 mm) and large size (diameter: 4.0 mm, length: 5.0 mm), respectively, by a G-250 granulating machine (Machine factory of South China University of Technology, Guangzhou, China). Pellets were steamed (90°C, 30 min), and then air-dried to about 100 g kg^−1^ moisture. Diets were stored with vacuum package (-20°C).

### 2.3. Feeding Trial and Experimental Conditions

An eight-week feeding experiment was conducted in aquarium of Ningbo University Meishan Campus (Ningbo, China). Juvenile mud crabs were obtained from mudflat in Xiangshan (Ningbo, China). Before experiment, potassium permanganate (KMnO_4_) was used to strictly disinfect the recirculating aquaculture system. Then, approximately 200 crabs were cultured in the vertical frame mounted recirculating aquaculture system for two weeks for acclimating to laboratory. Seawater was purified and sterilized through multistage filtration system with ultraviolet light. Light change, 12 hours light (8 : 00 to 20 : 00) and 12 hours dark, was set to mimic the alternation of day and night. Crabs were fed with commercial diet (460 g protein kg^−1^ diet and 80 g lipid kg^−1^ diet, Tongwei Co., Ltd.) during the acclimatization stage; and the feeding amount was 4%-6% of crab body weight. Robust body, uniform size, intact appendages, responsiveness to external stimuli, and disease-free were the selection criteria of healthy mud crab. Ultimately, 144 healthy and active crabs (initial weight 11.29 ± 0.09 g) were assigned into 144 aquaria (48.3 cm × 28.4 cm × 38 cm) to prevent the aggression of crab. Each experimental treatment was allocated for three replicates (*n* = 3), each replicate containing eight crabs. During the experimental period, crabs were fed at 18 : 00 with a feeding amount of 30 g diet kg^−1^ body weight to 60 g diet kg^−1^ body weight, which was adjusted by the actual consumptions and residual diets to keep an apparent satiation every day. The feces and residual diets were removed by siphon, and approximately one third of seawater was exchanged to keep the seawater clean and fresh. Water quality indicators were as follows: temperature 26.0 ± 1.30°C, salinity 22.70 ± 0.80 ppt, pH 7.05 ± 0.32, ammonia nitrogen < 0.05 mg L^−1^, nitrite nitrogen < 0.01 mg L^−1^, and dissolved oxygen 7.05 ± 0.42 mg L^−1^. Temperature and salinity were measured by electronic temperature and salinity meter (AZ Instrument Corp., Taiwan, China). Dissolved oxygen, pH, and ammonia nitrogen were measured by kits (Sunpu Biochemical and Technology Co., Ltd., Beijing, China).

### 2.4. Sample Collection

Crabs were fasted for one day and then anaesthetized in an ice-bath for five minutes until they were lightly anesthetized when feeding experiment ended. Crabs were counted and weighed to calculate survival, percentage weight gain (PWG), specific growth rate (SGR), and feed efficiency (FE) as well as molting ratio (MR). Hemolymph sample was drawn from pericardial cavity by 1 mL syringe, then sorted in 2 mL Eppendorf tube, and centrifuged using an Eppendorf centrifuge 5810R (1900 × *g*, 10 min). Supernatant was transferred to 200 *μ*L microcentrifuge tube, then stored (-80°C) until biochemical analysis (*n* = 3, per treatment). Hepatopancreas sample (about 100 mg per crab) was dissected and collected in 1.5 mL tube, quick-frozen in liquid nitrogen container, and then transferred to -80°C refrigerator. A small portion of hepatopancreas (50 mg per crab) was collected into 1.5 mL microfuge tube containing RNA keeper tissue stabilizer (Jiangsu Cowin Biotech Co., Ltd, Jiangsu, China), also quick-frozen in liquid nitrogen container, and then stored at -80°C for later gene expression analysis. Another small portion of hepatopancreas (about 100 mg per crab) was saved in fixative solution (4% paraformaldehyde, Beijing Solarbio Science & Technology Co., Ltd.) for the morphology analysis (*n* = 3). The remaining hepatopancreas and muscle (approximately 5 g) tissues were collected in 5 mL centrifugal tubes, respectively, and then stored at -80°C for measuring fatty acid composition and proximate composition (*n* = 3). All above tissue collection procedures were carried out quickly on ice to ensure the quality of samples.

### 2.5. Proximate Composition Analysis

Diet and tissue proximate compositions were measured by methods of Association of Official Analytical Chemists (AOAC) [24]. In short, moisture contents were measured by 105°C atmospheric pressure drying method. Crude protein contents were measured by Dumas combustion method with a FP-528 full-automatic protein analyzer (Leco, USA). Crude lipid contents were measured by Soxhlet method with a Soxtec System HT6 automatic Soxdahl solvant extractor (Tecator, Sweden). Ash contents were measured by a Sigma high-temperature muffle furnace (Shanghai, China) at 550°C for eight hours.

### 2.6. Hematological Characteristics Analysis

Contents of total protein (TP), triacylglycerol (TG), total cholesterol (T-CHO), glucose (GLU), alanine aminotransferase (ALT), aspartate aminotransferase (AST), and low-density lipoprotein cholesterol (LDL-C) as well as high-density lipoprotein cholesterol (HDL-C) were determined by a VITALAB Selectra Junior Pros automatic biochemistry analyzer (Netherlands) with BioSino Bio-Technology and Science analysis kits (Beijing, China).

### 2.7. Antioxidant Capacity Assays

Hepatopancreas samples were homogenized by a homogenizer (IKA® T25 digital Ultra-Turrax, Germany) with cold normal saline (0.86%) in 1 : 9 (*w* : *v*) on ice, and then centrifugated (1900 × *g*, 10 min, 4°C) using an Eppendorf centrifuge 5810R centrifuge (Germany). The supernatants were transferred into 200 *μ*L microcentrifuge tubes for the antioxidant capacity assays. Total antioxidant capacity (T-AOC), activities of total superoxide dismutase (T-SOD), catalase (CAT), and glutathione peroxidase (GSH-Px) as well as contents of reduced glutathione (GSH) and malondialdehyde (MDA) in hemolymph and hepatopancreas homogenates were determined using Nanjing Jiancheng Bioengineering kits (Nanjing, China). Hepatopancreas homogenates protein contents were measured by Biosharp BCA protein assay kits (Beijing, China).

T-AOC was determined using 2,2-azino-bis-3-ethylbenzothiazoline-6-sulfonic acid (ABTS) method [25]. ABTS was oxidized into green color with appropriate oxidant, and ABTS production would be inhibited in the presence of antioxidants. T-AOC could be measured by the absorbance of ABTS at 405 nm or 734 nm.

T-SOD activity was determined by hydroxylamine method using the diagnostic reagent kit. Briefly, reaction system of xanthine and xanthine oxidase generated superoxide anion radical, then reacted with hydroxylamine to produce nitric ion which can react with naphthalene diamine. Reaction product concentration was proportional to the amount of generated superoxide anion radical, which caused the increasing absorbance at 550 nm. For hemolymph, the corresponding SOD amount was one SOD activity unit (U) when the SOD inhibition rate per milliliter reached 50%. For tissue homogenate, the corresponding SOD amount was one SOD activity unit (U) when the SOD inhibition rate reached 50% per mg protein in 1 mL reaction solution. [26].

CAT activity was determined using kit by ammonium molybdate method. The decomposition of H_2_O_2_ by catalase was quickly stopped by the addition of ammonium molybdate, and the rest of H_2_O_2_ reacted with ammonium molybdate to form a faint-yellow complex. One-unit CAT activity was considered to be 1 mg hepatopancreas protein or 1 mL hemolymph-consumed 1 *μ*mol H_2_O_2_ at 405 nm for one second [27].

GSH-Px activity was measured by colorimetric method [28]. The activity of GSH-Px could be expressed by the speed of its enzymatic reaction that promoting the reaction of H_2_O_2_ with reduced glutathione (GSH) to produce H_2_O and oxidized glutathione. GSH-Px activity could be obtained by measuring the depletion of GSH in the enzymatic reaction.

GSH concentration was measured by previous method. Glutathione reacts with dithiodinitrobenzoic acid to form a yellow product with an absorbance of 420 nm [29].

The concentration of MDA was measured by thiobarbituric acid (TBA) method. Red product was formed by condensation of MDA and TBA in lipid peroxidation degradation products, with a maximum absorption peak at 532 nm [27].

### 2.8. Morphological Observation of Hepatopancreas

Fresh hepatopancreas samples were fastened in fixative solution and then made into paraffin sections as follows. The hepatopancreas samples were dissected quickly, and then were immersed in fixative solution. 24 hours later, the samples were trimmed and then dehydrated by ethanol (concentration increased from 75% to 100%). After that, samples were embedded by paraffin, then cut into 4 *μ*m sections. At last, the sections were stained by hematoxylin and eosin (H&E). Sections were taken photos by a Nikon Eclipse CI microscope (Tokyo, Japan). Besides, areas of restzellen as well as blasenzellen cells were determined using the software of Image Pro Plus 6.0, 10 measurements were obtained in each replicate.

### 2.9. Fatty Acid Composition Assays

Diet and tissue fatty acids absolute quantifications (mg g^−1^) were measured by previous method [30]. In brief, 1 mL internal standard solution (methyl tricosanoate, C23:0, 1 mg mL^−1^) was added into a 12 mL screwed glass tube. Then, lyophilized sample (100 mg) was added to the tube. Afterwards, 3 mL BHT solution (0.25 mg mL^−1^, 0.025 g BHT, 99 mL CH_3_OH, and 1 mL H_2_SO_4_) was also added. Later, the tube was vigorously shaken for 1 min by a vortex oscillator (Genius, IKA, Germany), and then incubated (80°C, 4 h) in a water bath kettle. After cooled down to room temperature, 1 mL *n*-hexane was added, then shacked by vortex oscillator for appropriately 1 min. Next, 1 mL ultrapure water was used for accelerating two-dimensional separation, and then centrifuged (1900 × *g*, 1 min) to eliminate bubbles. The supernatant was filtered through 0.22 *μ*m organic phase ultrafiltration membrane and transferred to a thread screw neck vial. Fatty acid methyl ester (FAME) solution was dried by a termovap sample concentrator in the vial, and the FAMEs were resuspended by 500 *μ*L *n*-hexane and then tested by a 7890B-5977A gas chromatography-mass spectrometry (GC-MS, Agilent Technologies, USA) equipped with a DB-WAX fused-silica ultrainert capillary column (30 m × 250 *μ*m internal diameter, film thickness 0.25 *μ*m, Agilent J&W Scientific, USA). Tissue and diet individual fatty acid concentrations were calculated by the ratios of FAME peak areas with C23:0 peak area. Final results were expressed as absolute concentration (mg g^−1^ dry matter).

### 2.10. Total RNA Extraction, Reverse Transcription, and Quantitative Real-Time PCR

Relative expression levels of lipid-metabolism genes were measured using real-time quantitative PCR (qPCR). RNA extraction, reverse transcription, and qPCR were conducted according to the previous method [31]. Briefly, hepatopancreas total RNA was extracted by a R401-01 RNA Isolater Total RNA Extraction Reagent (Vazyme, Nanjing, China). Quality of the extracted total RNA was tasted by denaturing agarose gel electrophoresis, and extracted total RNA concentration were determined by a Nanodrop 2000 ultramicro spectrophotometer (Thermo Fisher Scientific, USA). After that, extracted total RNA was reversed transcript to cDNA by a R223-01 HiScript® II Q RT SuperMix (Vazyme, Nanjing, China). Besides, qPCR specific primers (Supplementary Table 1) were designed based on cDNA sequences of corresponding genes in NCBI database by Primer3Plus webpage, and then synthesized by Tsingke Biotechnology Co., Ltd. (Beijing, China). Calibration curves were obtained from six cDNA sample individual dilution concentration gradients. Amplification efficiency formula was described as follows: *E* = 10^(−1/Slope)^ − 1, and all primers amplification efficiencies were appropriately equal (around 100%). PCR amplification was performed using a LightCycler 96 quantitative thermal cycler (Roche, Switzerland), with 0.4 *μ*L cDNA, 0.2 *μ*L primer, 5.0 *μ*L 2 × Q711 − 02 ChamQ Universal SYBR qPCR Master Mix (Vazyme, Nanjing, China), and 4.2 *μ*L RNase-free water. Thermal-cycling procedure was set as follows: 95°C for 2 min (heating stage), 95°C for 10 s (denaturation stage, 45 cycles), 58°C for 10 s (annealing stage), and 72°C for 20 s (extension phase). All gene relative expression levels were calculated by 2^–ΔΔCt^ method [32]. Additionally, *β*-actin was served as house-keeping gene, and 0.40 g kg^−1^ ARA treatment was regarded as reference group.

### 2.11. Statistical Analysis

Formulas involved in this study are described as follows:

(1) $$\begin{array}{c} \text{Percent weight gain }\left(\text{PWG},\%\right)=100\times \frac{\text{final body weight of crab }\left(\text{g}\right)–\text{initial body weight of crab }\left(\text{g}\right)}{\text{initial body weight of crab }\left(\text{g}\right),}, \end{array}$$

(2) $$\begin{array}{c} \text{Survival }\left(\%\right)=100\times \frac{\text{final number of crab}}{\text{initial number of crab}}, \end{array}$$

(3) $$\begin{array}{c} \text{Specific growth rate }\left(\text{SGR},\%\text{day}-1\right)=100\times \text{Ln }\frac{\text{final body weight of crab }\left(\text{g}\right)}{\text{initial body weight of crab }\left(\text{g}\right)/\text{days}}, \end{array}$$

(4) $$\begin{array}{c} \text{Feed efficiency }\left(\text{FE}\right)=\frac{\text{weight gain}\left(\text{g},\text{wet weight}\right)}{\text{feed consumed }\left(\text{g},\text{dry weight}\right)}, \end{array}$$

(5) $$\begin{array}{c} \text{Molting ratio}\left(\text{MR}\right)=\frac{2\times \text{molting number}}{\text{initial number of crab}+\text{final number of crab}}. \end{array}$$

The results were showed as the mean ± SD (*n* = 3). First, all data were tested to confirm normal distribution and variance homogeneity by the software of SPSS 22.0. Differences between mean values were analyzed using one-way analysis of variance (ANOVA) with Tukey's multiple range post hoc test (*p* < 0.05 considered as being significant). In addition, orthogonal polynomial contrasts were also applied to confirm linear and quadratic model significances. GraphPad Prism 8.0 software was applied to process histogram. Moreover, principal component analysis (PCA) was applied for multivariate data analysis of tissue fatty acid profiles by using the SIMCA (version P11, Umetrics, Sweden) software.

## 3. Results

### 3.1. Growth Performance and Feed Utilization

**Figure 1:**
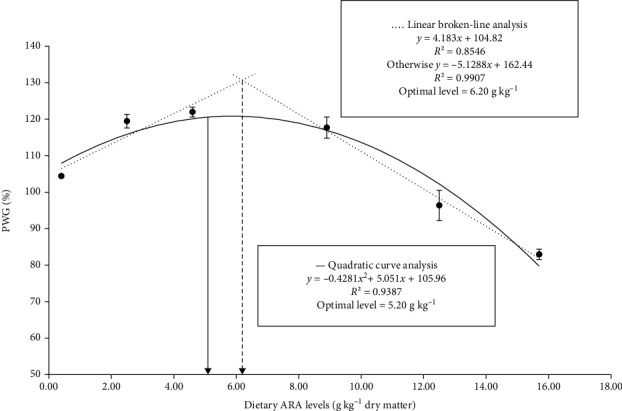
The linear broken-line model (dash line) and quadratic curve model (solid line) for the relationship between PWG and ARA levels in mud crab *Scylla paramamosain*.

Effects of ARA levels in diets on growth as well as feed utilization were presented in Table 3. Survival ranged from 74.83% to 87.50% among all treatments, and there were no significant differences among all treatments (*p* > 0.05). Crabs fed diets with 2.50, 4.60 and 8.90 g kg^−1^ ARA showed significantly higher PWG and SGR than those fed the other diets (*p* < 0.05), while there were no significant differences among these three treatments (*p* > 0.05). Crabs fed diet with 4.60 g kg^−1^ ARA exhibited significantly higher FE than those fed the other treatments (*p* < 0.05). Additionally, crabs fed diet with 4.60 g kg^−1^ ARA presented a significantly higher MR than those fed the other diets (*p* < 0.05), but no significant differences have been observed between 4.60 g kg^−1^ and 2.50 g kg^−1^ ARA groups (*p* > 0.05). Crabs fed diet with 4.60 g kg^−1^ ARA showed the highest PWG, SGR, FE, and MR among all groups. As presented in Figure 1, two-slope broken-line analysis and quadratic curve regression analysis of PWG against dietary ARA levels suggested that optimal dietary ARA levels were determined to be 5.20 and 6.20 g kg^−1^, respectively.

### 3.2. Proximate Composition in Hepatopancreas and Muscle

Impacts of ARA levels in diets on proximate composition in tissues were shown in Table 4. In hepatopancreas, lipid content showed an obvious downward trend with the increase of dietary ARA levels. Crabs fed diet with 0.40 g kg^−1^ ARA showed a significantly higher lipid content in hepatopancreas among all groups (*p* < 0.05). In muscle, lipid content also showed a similar trend to hepatopancreas in all treatments. Muscle lipid content in crab fed diet with 0.40 g kg^−1^ ARA presented the highest value in all groups (*p* < 0.05). Results also revealed that moisture, protein, and ash contents in hepatopancreas and muscle of mud crabs were not influenced by ARA levels in diets (*p* > 0.05).

### 3.3. Hematological Characteristics

Hematological characteristics are presented in Table 5. ALT and AST activities showed trends of decreasing first and then increasing with the increasing dietary ARA levels. Crabs fed diet with 15.70 g kg^−1^ ARA showed significantly higher ALT and AST activities among all treatments (*p* < 0.05). TG, T-CHO, and LDL-C contents showed obvious downward trends with the increase of dietary ARA levels. However, no significant differences in TP, GLU, and HDL-C contents have been found in all treatments (*p* > 0.05).

### 3.4. Oxidation and Antioxidant Parameters

**Figure 2:**
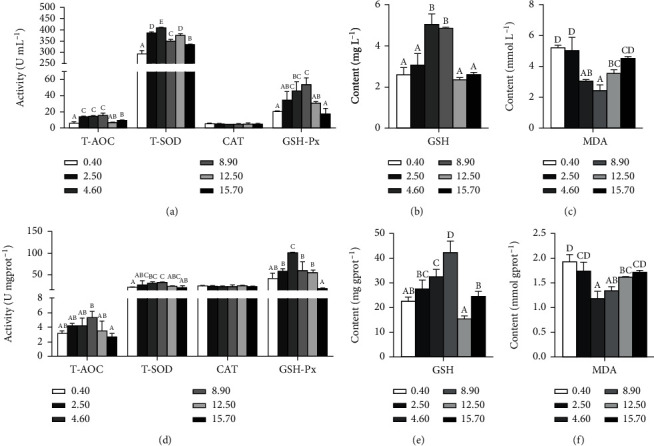
The antioxidant capacity of hemolymph (a–c) and hepatopancreas (d–f) of juvenile mud crab *Scylla paramamosain* fed with diets containing different dietary ARA levels. Values are mean (*n* = 3) with standard errors represented by vertical bars. Mean values for the same column with different letters were significantly different (*p* < 0.05). T-AOC: total antioxidant capacity; T-SOD: total superoxide dismutase; CAT: catalase; GSH-Px: glutathione peroxidase; GSH: reduced glutathione; and MDA: malondialdehyde.

Oxidation and antioxidant parameters in hemolymph and hepatopancreas were presented in Figure 2. In hemolymph, T-AOC and activities of T-SOD and GSH-Px increased first and then decreased with the increase of ARA levels in diets (Figure 2(a)). Crabs fed diets with 2.50, 4.60 and 8.90 g kg^−1^ ARA showed significantly higher T-AOC than those fed the other diets (*p* < 0.05). Crabs fed diet with 4.60 g kg^−1^ ARA showed the highest T-SOD activity in all groups (*p* < 0.05), and the lowest T-SOD activity was found in crab fed diet with 0.40 g kg^−1^ ARA (*p* < 0.05). The highest GSH-Px activity was observed in 8.90 g kg^−1^ ARA group, while the lowest GSH-Px activities were found in 0.40 and 15.70 g kg^−1^ ARA groups. CAT activity was not influenced by ARA levels in diets (*p* > 0.05). GSH concentration increased first and then decreased with the increase of dietary ARA levels (Figure 2(b)). The highest GSH contents were found in 4.60 and 8.90 g kg^−1^ ARA groups. MDA concentration decreased first and then increased with the increase of dietary ARA levels (Figure 2(c)). The lowest MDA content was found in 8.90 g kg^−1^ ARA group. In hepatopancreas, different dietary ARA levels significantly affected T-AOC and activities of T-SOD and GSH-Px (Figure 2(d)) (*p* < 0.05). Crabs fed with 8.90 g kg^−1^ ARA diet had a significantly higher T-AOC than those fed diet with 15.70 g kg^−1^ ARA (*p* < 0.05), while no significant differences were found at those fed the other diets (*p* > 0.05). The highest T-SOD activity was observed in 8.90 g kg^−1^ ARA group, which was significantly higher than those fed with 0.40 g kg^−1^ and 15.70 g kg^−1^ ARA diets (*p* < 0.05). Crabs fed with 4.60 g kg^−1^ ARA diet showed the highest GSH-Px activity in all groups (*p* < 0.05). CAT activity was not influenced by dietary ARA levels (*p* > 0.05). GSH concentration increased first and then decreased with the increase of dietary ARA levels (Figure 2(e)), and crabs fed diet with 8.90 g kg^−1^ ARA exhibited the highest GSH content among all treatments (*p* < 0.05). MDA concentration decreased first and then increased with the increase of dietary ARA levels (Figure 2(f)), and the lowest MDA content was found in crabs fed diet with 4.60 g kg^−1^ ARA.

### 3.5. Histology Observation in Hepatopancreas

**Figure 3:**
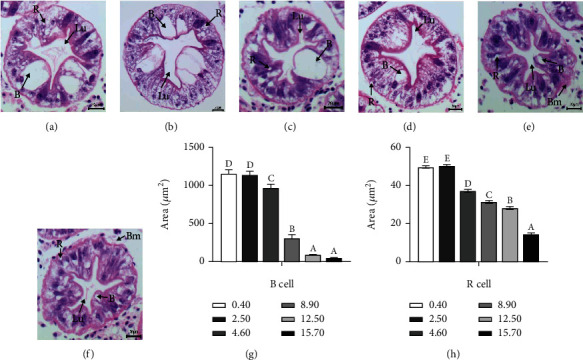
Effects of different dietary ARA levels on hepatopancreas histological structure of mud crab *Scylla paramamosain*. Histological sections of hepatopancreas are shown in (a) 0.40, (b) 2.50, (c) 4.60, (d) 8.90, (e) 12.50 and (f) 15.70 groups under 400× magnification, respectively. The areas of B cell (a) and R cell (b). Data were represented as mean ± SD of three replications (n =3). Different letters on the error bars indicate significant differences by Tukey's test (*P* <0.05). R cell, restzellen cell; B cell, blasenzellen cell; Lu, lumen structure; Bm, basement membrane.

Hepatopancreas histology were shown (Figures 3(a)–3(f)). Hepatopancreatic tubules were found relatively analogous with characteristic structures of epithelial cells, including blasenzellen cell (B) and restzellen cell (R), intact hepatopancreatic tubule lumen (Lu), and basement membrane (Bm), while the areas of B and R cells were significantly downregulated with the increasing ARA inclusions in diets (*p* < 0.05) (Figures 3(a) and 3(b)). Crabs fed with 0.40 g kg^−1^ and 2.50 g kg^−1^ ARA diets had significantly higher areas of B as well as R cells among all treatments (*p* < 0.05).

### 3.6. Fatty Acid Composition in Tissues

Tissue (hepatopancreas and muscle) fatty acid compositions are shown in Tables 6 and 7, respectively. Usually, ARA levels in diets significantly influenced tissue fatty acid profiles. In hepatopancreas, crabs fed diet with 15.70 g kg^−1^ ARA had significantly higher ARA deposition than other groups (*p* < 0.05). Gradual increases of SFA, MUFA, ALA, EPA, and DHA were found with the increasing dietary ARA levels. Besides, a significantly increasing was also observed in the ratio of ARA to EPA as well as the ratio of ARA to DHA (*p* < 0.05). In muscle, the fatty acid profile presented a similar trend to hepatopancreas. There was a positive correlation between ARA contents and dietary ARA levels. ARA content in 15.70 g kg^−1^ ARA group was significantly higher than other treatments (*p* < 0.05). Moreover, a significant increase in the ratio of ARA to EPA as well as the ratio of ARA to DHA were observed (*p* < 0.05), which was similar to hepatopancreas.

**Figure 4:**
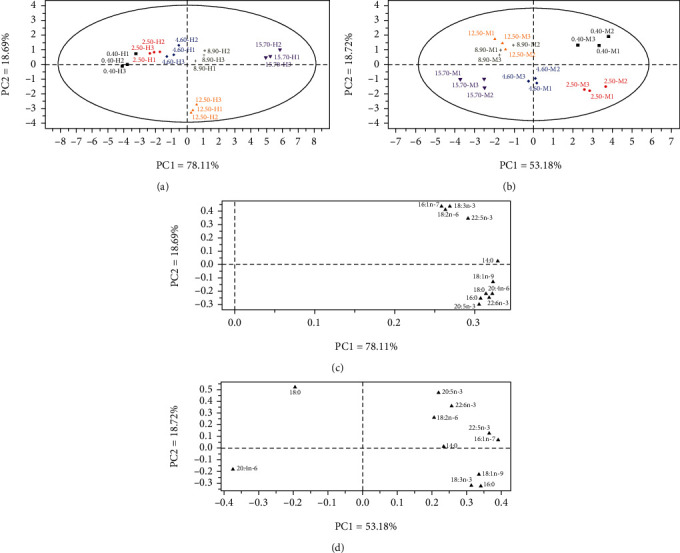
Principal component analysis (PCA) score plot (a, b) and loading plot (c, d) based on fatty acid compositions of hepatopancreas (a, c) and muscle (b, d) of juvenile mud crab *Scylla paramamosain* fed with different dietary ARA levels.

Principal component analysis (PCA) was used to offer an all-sided picture of hepatopancreas and muscle fatty acid profiles (Figure 4). The score plot of fatty acid profiles in hepatopancreas (Figure 4(a)) and the muscle (Figure 4(b)) showed the first two principal components occupied 96.80% (PC1, 78.11%; PC2, 18.69%) and 71.90% (PC1, 53.18%; PC2, 18.72%) variation in fatty acid profiles, respectively. PCA loading plot suggested that hepatopancreas and muscle individual fatty acid was in charge of the separation between treatments (Figures 4(c), 4(d)).

### 3.7. Expression Levels of Lipid-Metabolism Genes

**Figure 5:**
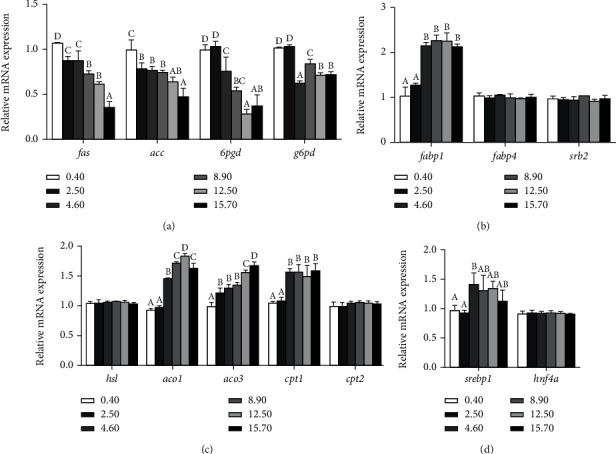
Effect of dietary ARA levels on relative mRNA expression levels of genes involved in lipid metabolism including anabolism (a), transport and uptake (b), catabolism (c), and transcription factors (d) in hepatopancreas of juvenile mud crab *Scylla paramamosain* fed with different dietary ARA levels. Significant differences at *p* < 0.05 (Tukey's test). fas: fatty acid synthase; acc: acetyl-CoA carboxylase; 6pgd: 6-phosphogluconate dehydrogenase; g6pd: glucose-6-phosphate dehydrogenase; fabp: fatty acid binding protein; srb2: scavenger receptor class 2; hsl: hormone-sensitive lipase; aco: 1-aminocyclop ropane-1-carboxylic acid oxidase; cpt: carnitine palmitoyltransferase; srebp1: sterol regulatory element-binding protein 1; and hnf4*α*: hepatocyte nuclear factor 4*α*.

Relative mRNA expression levels of genes related to lipid anabolism, transport and uptake, catabolism, and transcription factors were shown in Figure 5. It was showed that ARA levels in diets significantly affected the expression levels of lipid-metabolism genes (*p* < 0.05). Crabs fed with 0.40 g kg^−1^ ARA diet showed significantly higher relative gene expression levels of fas and acc than those fed the other diets (*p* < 0.05). Crabs fed diets with 0.40 and 2.50 g kg^−1^ ARA exhibited significantly higher expression levels of 6pgd and g6pd than those fed the other diets (*p* < 0.05) (Figure 5(a)). Gene expression level of fabp1 significantly increased when crabs fed diets with 4.60, 8.90, 12.50, and 15.70 g kg^−1^ ARA (*p* < 0.05). However, gene expression levels of fabp4 and srb2 were not influenced by dietary ARA levels (*p* > 0.05) (Figure 5(b)). Crabs fed with 12.50 g kg^−1^ diet presented a significantly higher expression level of aco1 among all treatments (*p* < 0.05). In addition, the expression level of aco3 significantly upregulated with the increase of dietary ARA levels (*p* < 0.05). Crabs fed diets with 4.60, 8.90, 12.50, and 15.70 g kg^−1^ ARA showed significantly higher expression levels of cpt1 than those fed with 0.40 and 2.50 g kg^−1^ ARA diets (*p* < 0.05). However, expression levels of hsl and cpt2 were not influenced by ARA contents in diets (*p* > 0.05) (Figure 5(c)). Relative expression level of transcription factor indicated that srebp1 significantly upregulated in 4.60 g kg^−1^ group than those fed diets with 0.40 and 2.50 g kg^−1^ ARA, while no statistical differences were found among 4.60, 8.90, 12.50, and 15.70 g kg^−1^ (*p* > 0.05). And no significant differences were observed in the expression level of hnf4*α* among all groups (*p* > 0.05) (Figure 5(d)).

## 4. Discussion

Generally, dietary ARA has been investigated to participate in promoting growth performance of various aquatic animals, such as blue gourami *Trichopodus trichopterus* [33], javelin goby *Synechogobius hasta* [10], Japanese eel *Anguilla japonica* [34], Japanese seabass *Lateolabrax japonicus* [13], yellow catfish *Pelteobagrus fulvidraco* [11], and oriental river prawn *Macrobrachium nipponense* [16]. However, some studies reported that growth parameters did not differ between different ARA groups, such as blue gourami *Trichopodus trichopterus* [12] and gilthead sea bream *Sparus aurata* [35]. Even, dietary ARA inclusion resulted in negative impacts on growth performance, including Pacific white shrimp *Litopenaeus vannamei* [36]. In this study, survival ranged from 74.83% to 87.50% in all treatments, and no significant differences were found among all treatments. Analogously, survival was not significantly affected by different dietary ARA inclusions in postlarva of mud crab *Scylla paramamosain* [23]. Similar results also indicated that there was no significant differences in survival among all ARA groups in Chinese mitten crab *Eriocheir sinensis* [20]. Additionally, a few studies also demonstrated that the survivals of some aquatic animal have not been significantly influenced by various dietary ARA levels, including juvenile oriental river prawns *Macrobrachium nipponense* [16], juvenile Japanese seabass *Lateolabrax japonicus* [13], and Malabar red snapper *Lutjanus malabaricus* fingerlings [37]. In this study, crabs fed with 2.50 g kg^−1^, 4.60 g kg^−1^, and 8.90 g kg^−1^ ARA diets had significantly higher PWG and SGR than other groups, while no significant differences were found among these three groups. Two-slope broken-line analysis and quadratic curve regression analysis of PWG against ARA levels in diets revealed that optimal dietary ARA levels were 5.20 g kg^−1^ and 6.20 g kg^−1^ for juvenile mud crab. Similarly, PWG and SGR of mud crab *Scylla paramamosain* postlarva (initial weight 8.15 mg) were significantly improved when dietary ARA level increased to 11.80 g ARA kg^−1^ diet, and then decreased with more ARA supplementation in diets [23]. Probable reason for the different optimal dietary ARA levels is that crabs are at different growth stages. Thus, this study provides an important reference for the ARA requirements of juvenile mud crabs. Besides, Chinese mitten crab *Eriocheir sinensis* (initial weight 1.01 ± 0.12 g) in the group of 23.70 g ARA kg^−1^ total lipid content had the highest PWG and SGR among all groups [20]. The different research results may be caused by the species-specific responses or different growth stages. Besides, crabs fed with 4.60 g kg^−1^ ARA diet showed a significantly higher FE than other treatments. Differently, dietary ARA treatments did not influence FE of Malabar red snapper *Lutjanus malabaricus* fingerlings [37]. The different results may be caused by the species-specific responses or the different concentrations of dietary ARA. Additionally, in this study, crabs fed with 4.60 g kg^−1^ ARA diet presented a significantly higher MR than 0.40 g kg^−1^, 8.90 g kg^−1^, 12.50 g kg^−1^, and 15.70 g kg^−1^ groups. Differently, no significant differences have been found in MR between dietary ARA groups in postlarva of mud crab *Scylla paramamosain* [23]. These results further indicated that ARA nutrition played a key role in the molting process of juvenile mud crab.

Hemolymph metabolites of crustaceans represent indicators of physiological, nutritional, and immune stress [38]. ALT as well as AST are served as important indicators for diagnosis of hepatopancreas function [39]. In this study, ALT and AST activities in hemolymph showed obvious trends of decreasing first and then increasing with the increasing ARA levels in diets, which indicated that moderate ARA level in diet was beneficial to hepatopancreas function of mud crab. Similar results were reported that juvenile yellow catfish *Pelteobagrus fulvidraco* fed with the diets of 49.60 ARA kg^−1^ total fatty acids and 74.90 ARA kg^−1^ total fatty acids presented lower AST activity in serum than other groups; and the highest AST activity was found in the group of 3.90 ARA kg^−1^ total fatty acids [11]. Another research on juvenile grass carp *Ctenopharyngodon idellus* showed that non-ARA-supplemented group showed higher ALT and AST activities in serum than ARA-added groups [40]. Meanwhile, ALT and AST activities in 15.70 g kg^−1^ ARA group were the highest among all treatments, which indicated that excessive dietary ARA levels resulted in negative impacts on health of mud crab. In addition, TG, T-CHO, and LDL-C contents showed obvious downward trends with the increasing ARA levels in diets, which demonstrated that ARA supplementation in diets could reduce the lipid content of hemolymph.

Aerobic animals are able to produce reactive oxygen species (ROS) in cellular metabolic process, which will lead to cell and tissue damages [41]. Crabs possess some physiological methods to eliminate oxidative stress, including T-SOD, CAT, T-AOC, and GSH [42, 43]. In the present study, T-AOC, T-SOD, and GSH-Px activities as well as GSH contents in hemolymph and hepatopancreas increased first and then decreased approximately with the increasing ARA levels in diets, which demonstrated that moderate ARA addition in diets was able to enhance antioxidant capacity in mud crab. It was probably caused by that the eicosanoids derived from ARA directly participated in immunoregulation [4]. Similarly, SOD as well as T-AOC activities increased with dietary ARA inclusion in postlarva of mud crab *Scylla paramamosain* [23]. Researchers have also found that dietary ARA could significantly enhance SOD and GSH-Px activities in Chinese mitten crab *Eriocheir sinensis* [19]. Results were reported in javelin goby *Synechogobius hasta* which revealed that GSH-Px and CAT activities in liver increased with the increasing ARA levels in diets [10].

Hepatopancreas is a crucial organ for nutrient absorption and storage in crustaceans, which plays crucial roles in the absorption and storage of metabolic substrates and the synthesis and secretion of digestive enzymes [44]. In general, R cell, the most abundant cell in hepatopancreas, is responsible for storing lipid and glycogen. While, B cell is in charge of intracellular digestion with secretory function. In this study, areas of B and R cells presented obvious downward trends with the increasing dietary ARA levels. The reduction of lipid deposition in hepatopancreas treated with ARA diet may be responsible for vacuolation, compression and atrophy of B and R cells. Therefore, hepatopancreas histology is a good indicator of hepatopancreas lipid content, further supporting the notion that dietary ARA supplementation is able to reduce hepatopancreas lipid deposition. Previous study also revealed that dietary ARA inclusion reduced lipid deposition on hepatopancreatic cells [36].

Generally, tissue fatty acid profiles reflected diet fatty acids compositions. In hepatopancreas, ARA contents increased with the increasing dietary ARA levels. Similarly, Pacific white shrimp *Litopenaeus vannamei* juveniles fed with 0.6% diet presented higher ARA deposition in hepatopancreas compared to the others experimental groups [36]. Dietary ARA significantly increased ARA amount in liver of juvenile grass carp *Ctenopharyngodon idellus* [45]. Increasing ARA content in liver was observed in juvenile javelin goby *Synechogobius hasta* fed with the diet containing increasing dietary ARA levels [10]. ARA proportion in liver of juvenile yellow catfish *Pelteobagrus fulvidraco* significantly increased with dietary ARA levels increasing from 0.39% to 12.64% of total fatty acids [11]. EPA concentrations also increased with the increasing dietary ARA levels. However, liver EPA levels in juvenile Japanese seabass *Lateolabrax japonicus* were inversely related to dietary ARA levels [13]. EPA proportion in liver of juvenile yellow catfish *Pelteobagrus fulvidraco* significantly decreased with increase of dietary ARA levels [11]. DHA concentrations also increased with the increasing dietary ARA levels. Differently, a higher DHA accumulation was revealed in the control compared to the rest of the experimental treatments in hepatopancreas [36]. ∑SFA, ∑MUFA, ∑n − 6 PUFA, ∑n − 3 PUFA, and ∑n − 3 LC − PUFA significantly increased with the increasing dietary ARA levels. In muscle, ARA contents also increased with the increasing dietary ARA levels. EPA contents significantly decreased with the increasing dietary ARA levels, while ARA and EPA contents in muscle of Chinese mitten crab *Eriocheir sinensis* increased first and then decreased with the dietary ARA level increased [20]. The different results may be caused by different species, which reflected the species specificity of fatty acids profiles. DHA levels approximately decreased with the increasing dietary ARA levels. ∑SFA contents increased first and then decreased with dietary increasing ARA levels. ∑MUFA contents decreased with the increasing dietary ARA levels. ∑n − 6 PUFA contents significantly increased with the increased dietary ARA levels, which was mainly caused by the increasing ARA contents in muscle. The different fatty acid profiles in hepatopancreas and muscle described above reflected the tissue specificity of fatty acid compositions; and fatty acids usually performed different functions in different tissues. Moreover, the PCA plots of fatty acid in tissues showed a more significant effects of different dietary ARA levels on fatty acid composition in hepatopancreas than that in muscle. From the distances of the points on Figure 4(a), 12.50 g kg^−1^ and 15.70 g kg^−1^ ARA groups were far away from other treatments, while the distances of the points on Figure 4(b) were approximately equally distributed. This indicates that the hepatopancreas, as a metabolic organ of crustaceans, has significant differences in response to the dietary ARA levels.

In this study, hepatopancreas and muscle lipid contents significantly downregulated with the increasing ARA concentrations in diets. Similarly, studies have been reported in gilthead sea bream *Sparus aurata* L. [35] as well as Japanese seabass *Lateolabrax japonicus* [13], which revealed that whole body, muscle, and gill showed decreasing lipid contents with the increasing dietary ARA levels. ARA and their metabolites may be important regulators of PPAR*γ*, which may affect the transcription of genes in lipid metabolism, and thereafter regulate fatty acid synthesis and storage [46]. However, PPAR*γ* has not been found in crustaceans so far. On the other hand, ARA probably participated in the regulating lipid metabolism pathway [40]. Previous studies have shown that fatty acid synthetase (fas) could catalyze continuous condensation reactions to produce fatty acids and was critical in lipid homeostasis [10]. Acetyl-CoA carboxylase (acc), 6-phosphogluconate dehydrogenase (6pgd) as well as glucose-6-phosphate dehydrogenase (g6pd) are able to regulate biosynthesis of fatty acids. As a major tissue of lipid storage, hepatopancreas plays critical roles in lipid metabolism [35]. In this experiment, relative expression levels of fas, acc, 6pgd as well as g6pd decreased with the increasing ARA levels in diets, which demonstrated that dietary ARA was able to reduce lipid anabolism and further decrease lipid deposition in hepatopancreas. Another study on yellow catfish *Pelteobagrus fulvidraco* revealed that gene expression levels of acc*α*, g6pd and 6pgd in liver decreased significantly with ARA levels in diets increasing from 3.90 g ARA kg^−1^ total fatty acids to 126.40 g ARA kg^−1^ total fatty acids [11]. Additionally, carnitine palmitoyl transferase 1 (cpt1) is a mitochondrial enzyme, which forms acyl carnitines by catalyzing transfer the acyl group of acyl-CoA from CoA to L-carnitine, which is a rate-limiting step in fatty acid oxidation [47]. Expression levels of lipid-catabolism genes have been also influenced by dietary ARA levels, including hsl, cpt1, aco1, and aco3. Relative expression levels of hsl, cpt1, aco1, and aco3 upregulated with the increasing dietary ARA levels and then downregulated hepatopancreas lipid deposition. Besides, fatty acids are transported and stored into mitochondria by fatty acid binding protein (fabp) [48]. Scavenger receptor class 2 (srb2) is able to regulate uptake rate of cellular fatty acids in mitochondrial membrane [49]. Srb2 is related to cpt1 on mitochondrial membrane and is able to improve function of CPT1 [50]. In this research, relative expression level of fabp1 upregulated with the increasing ARA levels in diets, which indicated that dietary ARA was able to promote fatty acid transport and absorption. Similar results showed that the expression level of fabp7 (involved in fatty acid transport as well as uptake) in European sea bass juveniles *Dicentrarchus labrax* increased in liver of fish in the group of 0.10 g ARA g^−1^ diet [51]. In crustaceans, relative expression levels of lipid-metabolism genes are regulated by transcription factor, such as sterol regulatory element-binding protein (srebp) and hepatocyte nuclear factor 4*α* (hnf4*α*). SREBP is involved in regulating genes related to fatty acids, cholesterol, phospholipids, and triglyceride biosynthesis [52]. In this experiment, relative expression level of srebp1 was first upregulated and then downregulated with the increasing ARA levels in diets. And the highest expression level of srebp1 was found in 4.60 g kg^−1^ ARA group. Above results reveled that lipogenesis was restrained with the increasing ARA levels in diets. Besides, further studies are needed to elucidate regulatory mechanism of the dietary ARA in mud crab.

## 5. Conclusion

Optimum dietary ARA levels can promote growth, enhance antioxidant capacity, and improve health of mud crab juveniles. According to two-slope broken-line and quadratic curve regression analysis of PWG against dietary ARA levels, optimal dietary ARA levels were determined to be 5.20 g kg^−1^ and 6.20 g kg^−1^, respectively. Excess dietary ARA levels had negative effects on the growth and health of juvenile mud crab. It also demonstrated that lipogenesis has been restrained with the increasing dietary ARA levels. These findings could provide theoretical guidance and reference for the lipid nutrition requirement research as well as the development of the commercial diet in juvenile mud crab.

## Figures and Tables

**Table 1:** Ingredients and proximate composition of the experimental diets (g kg^−1^ dry matter).

Ingredients	Dietary ARA levels (g kg^−1^)					
	0.40	2.50	4.60	8.90	12.50	15.70
Peru fish meal^a^	330.0	330.0	330.0	330.0	330.0	330.0
Soybean protein concentrate^a^	260.0	260.0	260.0	260.0	260.0	260.0
Yeast extract^a^	30.0	30.0	30.0	30.0	30.0	30.0
Krill meal^a^	30.0	30.0	30.0	30.0	30.0	30.0
Wheat flour^a^	238.0	238.0	238.0	238.0	238.0	238.0
ARA-enriched oil (50% ARA)^b^	0.0	5.0	10.0	20.0	30.0	40.0
Palmitic acid^b^	40.0	35.0	30.0	20.0	10.0	0.0
Soybean lecithin^c^	10.0	10.0	10.0	10.0	10.0	10.0
Cholesterol	2.0	2.0	2.0	2.0	2.0	2.0
Ca (H_2_PO_4_)_2_	5.0	5.0	5.0	5.0	5.0	5.0
Vitamin premix^d^	10.0	10.0	10.0	10.0	10.0	10.0
Mineral premix^e^	20.0	20.0	20.0	20.0	20.0	20.0
Choline chloride	3.0	3.0	3.0	3.0	3.0	3.0
Sodium alginate	20.0	20.0	20.0	20.0	20.0	20.0
BHT^f^	2.0	2.0	2.0	2.0	2.0	2.0
Proximate composition (g kg^−1^ dry matter)						
Moisture	99.6	95.1	91.8	91.9	98.9	96.5
Crude protein	484.9	488.4	481.0	488.5	489.7	483.4
Crude lipid	78.1	77.9	76.7	78.3	76.0	75.0
Ash	102.5	101.8	102.4	102.5	100.9	100.4

^a^Peru fish meal (g kg^−1^ dry matter): crude protein 707.7, crude lipid 87.8; Soybean protein concentrate (g kg^−1^ dry matter): crude protein 690.9, crude lipid 4.3; yeast extract (g kg^−1^ dry matter): crude protein 452.7, crude lipid 7.8; krill meal (g kg^−1^, dry matter): crude protein 543.3, crude lipid 211.3; and wheat flour (g kg^−1^ dry matter): crude protein 161.2, crude lipid 14.1. These ingredients were purchased from Ningbo Tech-Bank Feed Co., Ltd. (Ningbo, China). ^b^ARA-enriched oil and palmitic acid were purchased from Changsha Kenan Biotechnology Co., Ltd. (Changsha, China).^c^Soybean lecithin was purchased from Ningbo Tech-Bank Feed Co., Ltd. (Ningbo, China). ^d^Vitamin premix (IU or g kg^−1^ premix): retinyl acetate 2,500,000 IU; cholecalciferol 500,000 IU; all-rac-a-tocopherol 25,000 IU; menadione 5.63 g; thiamine 11.25 g; riboflavin 9.5 g; ascorbic acid 95 g; pyridoxine hydrochloride 10 g; cyanocobalamin 0.02 g; folic acid 2 g; biotin 0.375 g; nicotinic acid 37.5 g; D-calcium pantothenate 21.5 g; inositol 80 g; and ethoxyquin (antioxidant) 0.5 g. All ingredients were diluted with corn starch to 1 kg. ^e^Mineral mixture (g kg^−1^ premix): FeC_6_H_5_O_7_ 4.57 g; ZnSO_4_·7H_2_O 9.43 g; MnSO_4_·H_2_O (99%) 4.14 g; CuSO_4_·5H_2_O (99%) 6.61 g; MgSO_4_·7H_2_O (99%) 238.97 g; KH_2_PO_4_ 233.2 g; NaH_2_PO_4_ 137.03 g; C_6_H_10_CaO_6_·5H_2_O (98%) 34.09 g; and CoCl_2_·6H_2_O (99%) 1.36 g. All ingredients were diluted with corn starch to 1 kg. ^f^BHT was purchased from Aladdin Industrial Co., Ltd. (Shanghai, China).

**Table 2:** Fatty acid compositions of different experimental diets (mg g^−1^ dry matter).

Fatty acids	Dietary ARA levels (g kg^−1^)					
	0.40	2.50	4.60	8.90	12.50	15.70
14 : 0	2.52	2.31	2.30	2.35	2.37	2.41
16 : 0	31.43	26.73	24.96	21.17	15.27	9.50
18 : 0	2.08	2.09	2.35	2.85	3.26	3.65
∑*SFA*^*a*^	36.03	31.14	29.61	26.37	20.91	15.56
16:1n-7	3.04	2.79	2.80	2.83	2.84	2.86
18:1n-9	8.41	8.10	8.53	9.40	9.92	10.45
20:1n-9	0.26	0.25	0.28	0.33	0.36	0.40
∑*MUFA*^*b*^	11.71	11.14	11.62	12.57	13.13	13.70
18:2n-6	3.81	3.71	3.94	4.29	4.34	4.58
20:4n-6	0.43	2.54	4.64	8.92	12.54	15.73
∑*n* − 6*PUFA*^*c*^	4.24	6.25	8.58	13.21	16.88	20.31
18:3n-3	1.01	0.94	0.99	1.03	1.01	1.04
18:4n-3	0.56	0.50	0.52	0.53	0.53	0.54
20:5n-3	5.56	5.19	5.13	5.24	5.39	5.36
22:5n-3	0.75	0.72	0.71	0.71	0.73	0.74
22:6n-3	4.71	4.34	4.35	4.31	4.17	3.87
∑*n* − 3*PUFA*^*d*^	12.59	11.69	11.71	11.82	11.83	11.55
n-3/n-6 PUFA^e^	2.97	1.87	1.36	0.89	0.70	0.57
EPA/DHA^f^	1.18	1.20	1.18	1.22	1.29	1.39
ARA/EPA^g^	0.08	0.49	0.90	1.70	2.33	2.93
ARA/DHA^h^	0.09	0.58	1.07	2.07	3.01	4.07

Some fatty acids, of which the contents are minor, trace amount or not detected, such as 12 : 0, 20 : 0, 22 : 0, 24 : 0, 22:1n-9, 22:1n-11, 20:2n-6, 20:3n-6, 22:4n-6, 20:5n-6, 20:3n-3, and 20:4n-3, were not listed in this table. ^a^SFA: saturated fatty acids; ^b^MUFA: monounsaturated fatty acids; ^c^n-6 PUFA: n-6 polyunsaturated fatty acids; ^d^n-3 PUFA: n-3 polyunsaturated fatty acids; ^e^n-3/n-6 PUFA: n-3 polyunsaturated fatty acids: n-6 polyunsaturated fatty acids; ^f^EPA/DHA: the ratio of EPA to DHA; ^g^ARA/EPA: the ratio of ARA to EPA; and ^h^ARA/DHA: the ratio of ARA to DHA.

**Table 3:** Growth performance and feed utilization of juvenile mud crab *Scylla paramamosain* fed with different dietary ARA levels.

Items	Dietary ARA levels (g kg^−1^)						ANOVA	Linear		Quadratic	
	0.40	2.50	4.60	8.90	12.50	15.70	*p* value	*p* value	*R* ^2^	*p* value	*R* ^2^
IBW (g)	11.36 ± 0.21	11.26 ± 0.01	11.58 ± 0.04	11.36 ± 0.05	11.02 ± 0.17	11.17 ± 0.06	0.074	0.119	0.092	0.127	0.139
PWG (%)	104.41 ± 0.32^c^	119.47 ± 1.86^d^	121.98 ± 1.34^d^	117.71 ± 2.89^d^	96.38 ± 4.16^b^	82.95 ± 1.41^a^	0.000	0.002	0.431	0.000	0.875
Survival (%)	74.83 ± 7.22	75.00 ± 0.00	87.50 ± 0.00	83.33 ± 7.22	79.17 ± 7.22	75.00 ± 12.50	0.127	0.686	0.051	0.037	0.269
SGR (% day^−1^)	1.28 ± 0.00^c^	1.40 ± 0.02^d^	1.42 ± 0.01^d^	1.39 ± 0.02^d^	1.20 ± 0.04^b^	1.08 ± 0.01^a^	0.000	0.001	0.445	0.000	0.887
FE	0.67 ± 0.01^c^	0.75 ± 0.01^d^	0.80 ± 0.01^e^	0.72 ± 0.03^d^	0.64 ± 0.01^b^	0.55 ± 0.02^a^	0.000	0.003	0.392	0.000	0.804
MR	0.49 ± 0.04^ab^	0.57 ± 0.08^bc^	0.71 ± 0.04^c^	0.50 ± 0.05^ab^	0.46 ± 0.03^ab^	0.38 ± 0.04^a^	0.012	0.037	0.197	0.012	0.368

Values are represented as the mean ± SD of three replicates (*n* = 3). Values in the same row with different superscripts are significantly different (*p* < 0.05). IBW: initial body weight; PWG: percent weight gain; SGR: specific growth rate; FE: feed efficiency; and MR: molting ratio.

**Table 4:** Proximate compositions (%, wet weight) of hepatopancreas and muscle in juvenile mud crab *Scylla paramamosain* fed with different dietary ARA levels.

Items	Dietary ARA levels (g kg^−1^)						ANOVA	Linear		Quadratic	
	0.40	2.50	4.60	8.90	12.50	15.70	*p* value	*p* value	*R* ^2^	*p* value	*R* ^2^
*Hepatopancreas*											
Moisture	72.56 ± 0.06	72.75 ± 0.10	72.63 ± 0.04	72.66 ± 0.07	72.71 ± 0.09	72.51 ± 0.11	0.390	0.676	0.051	0.282	0.043
Crude protein	11.60 ± 0.12	11.65 ± 0.03	11.52 ± 0.29	11.55 ± 0.14	11.58 ± 0.12	11.53 ± 0.12	0.190	0.349	0.004	0.262	0.052
Crude lipid	11.37 ± 0.17^d^	10.68 ± 0.27^c^	10.07 ± 0.01^bc^	9.53 ± 0.01^b^	9.48 ± 0.04^b^	8.58 ± 0.01^a^	0.000	0.000	0.921	0.000	0.920
Ash	1.52 ± 0.67	1.51 ± 0.48	1.53 ± 0.55	1.60 ± 0.23	1.58 ± 0.41	1.53 ± 0.41	0.737	0.366	0.008	0.512	0.036
*Muscle*											
Moisture	79.53 ± 1.19	79.60 ± 1.55	79.50 ± 1.10	79.61 ± 1.18	79.67 ± 1.50	79.61 ± 1.89	0.960	0.485	0.030	0.790	0.098
Crude protein	16.55 ± 0.62	16.56 ± 0.88	16.58 ± 1.33	16.43 ± 0.58	16.58 ± 0.94	16.63 ± 1.38	0.787	0.656	0.049	0.645	0.069
Crude lipid	0.86 ± 0.29^e^	0.72 ± 0.17^d^	0.66 ± 0.2^cd^	0.60 ± 0.09^bc^	0.52 ± 0.18^ab^	6^a^	0.000	0.000	0.905	0.000	0.943
Ash	2.89 ± 0.50	2.92 ± 0.35	2.91 ± 0.26	2.95 ± 0.20	2.89 ± 0.32	2.87 ± 0.48	0.698	0.710	0.053	0.343	0.017

Data are reported as the mean ± SD of three replicates (*n* = 3). Values within the same row with different superscripts are significantly different (*p* < 0.05).

**Table 5:** Hematological characteristics of juvenile mud crab *Scylla paramamosain* fed with different dietary ARA levels.

Items	Dietary ARA levels (g kg^−1^)						ANOVA	Linear		Quadratic	
	0.40	2.50	4.60	8.90	12.50	15.70	*p* value	*p* value	*R* ^2^	*p* value	*R* ^2^
TP (g L^−1^)	53.86 ± 2.10	55.85 ± 3.98	52.82 ± 1.39	55.6 ± 0.17	53.78 ± 0.91	52.86 ± 1.94	0.848	0.627	0.046	0.769	0.094
GLU (g L^−1^)	1.53 ± 0.01	1.53 ± 0.14	1.48 ± 0.03	1.55 ± 0.02	1.43 ± 0.01	1.62 ± 0.14	0.670	0.686	0.051	0.611	0.061
TG (mmol L^−1^)	0.26 ± 0.02^c^	0.21 ± 0.01^bc^	0.16 ± 0.02^ab^	0.15 ± 0.01^a^	0.13 ± 0.00^a^	0.13 ± 0.01^a^	0.000	0.000	0.628	0.000	0.773
T-CHO (mmol L^−1^)	0.31 ± 0.02^c^	0.27 ± 0.02^c^	0.23 ± 0.01^b^	0.22 ± 0.01^b^	0.19 ± 0.01^b^	0.16 ± 0.01^a^	0.000	0.000	0.792	0.000	0.802
ALT (U L^−1^)	129.19 ± 3.39^c^	97.57 ± 0.24^b^	55.13 ± 4.13^a^	93.07 ± 1.57^b^	108.93 ± 0.58^bc^	220.59 ± 13.93^d^	0.000	0.014	0.282	0.000	0.898
AST (U L^−1^)	133.25 ± 0.33^b^	97.65 ± 0.90^a^	77.83 ± 2.40^a^	102.39 ± 0.92^a^	144.3 ± 14.25^b^	252.77 ± 6.32^c^	0.000	0.001	0.462	0.000	0.959
HDL-C (mmol L^−1^)	0.15 ± 0.01	0.15 ± 0.02	0.15 ± 0.01	0.16 ± 0.01	0.13 ± 0.01	0.14 ± 0.00	0.468	0.298	0.009	0.369	0.008
LDL-C (mmol L^−1^)	0.21 ± 0.01^b^	0.17 ± 0.01^*ab*^	0.15 ± 0.01^*ab*^	0.13 ± 0.02^*a*^	0.13 ± 0.02^*a*^	0.12 ± 0.01^*a*^	0.004	0.000	0.580	0.000	0.652

Data are presented as the mean ± SD of three replicates (*n* = 3). Values in the same column with different superscripts are different (*p* < 0.05). TP: total protein; GLU: glucose; TG: triglyceride; T-CHO: total cholesterol; ALT: alanine aminotransferase; AST: aspartate aminotransferase; HDL-C: high-density lipoprotein-cholesterol; and LDL-C: low-density lipoprotein-cholesterol.

**Table 6:** Hepatopancreas fatty acid composition of juvenile mud crab *Scylla paramamosain* fed with different dietary ARA levels (mg g^−1^ dry matter).

Fatty acids	Dietary ARA levels (g kg^−1^)						ANOVA	Linear		Quadratic	
	0.40	2.50	4.60	8.90	12.50	15.70	*p* value	*p* value	*R* ^2^	*p* value	*R* ^2^
14 : 0	1.55 ± 0.16^a^	2.13 ± 0.11^ab^	2.47 ± 0.18^b^	2.70 ± 0.15^b^	2.7 ± 0.05^b^	3.91 ± 0.31^c^	0.000	0.000	0.740	0.000	0.728
16 : 0	18.39 ± 0.22^a^	20.22 ± 0.34^b^	22.11 ± 0.32^c^	23.55 ± 0.17^d^	25.47 ± 0.22^e^	26.59 ± 0.17^e^	0.000	0.000	0.954	0.000	0.971
18 : 0	4.26 ± 0.32^a^	4.24 ± 0.13^a^	5.43 ± 0.10^b^	7.29 ± 0.23^c^	8.48 ± 0.16^d^	10.12 ± 0.00^e^	0.000	0.000	0.969	0.000	0.971
∑*SFA*^*a*^	24.20 ± 0.22^a^	26.59 ± 0.57^b^	30.01 ± 0.24^c^	33.54 ± 0.22^d^	36.66 ± 0.20^e^	40.62 ± 0.43^f^	0.000	0.000	0.984	0.000	0.984
16:1n-7	2.21 ± 0.38^a^	3.02 ± 0.13^ab^	3.40 ± 0.21^bc^	4.14 ± 0.41^cd^	4.40 ± 0.10^d^	5.57 ± 0.18^e^	0.000	0.000	0.848	0.000	0.838
18:1n-9	12.92 ± 0.42^a^	13.51 ± 0.33^a^	14.20 ± 0.47^a^	18.16 ± 0.19^b^	21.55 ± 0.20^c^	30.56 ± 0.09^d^	0.000	0.000	0.879	0.000	0.977
∑*MUFA*^*b*^	15.13 ± 0.19^a^	16.53 ± 0.44^b^	17.40 ± 0.45^b^	22.30 ± 0.53^c^	25.95 ± 0.11^d^	36.13 ± 0.27^e^	0.000	0.000	0.902	0.000	0.974
18:2n-6	2.84 ± 0.11^a^	4.29 ± 0.04^b^	4.90 ± 0.29^bc^	5.41 ± 0.15^c^	6.50 ± 0.20^d^	8.25 ± 0.56^e^	0.000	0.000	0.890	0.000	0.884
20:4n-6	1.46 ± 0.04^a^	2.94 ± 0.03^b^	6.26 ± 0.10^c^	12.24 ± 0.04^d^	18.09 ± 0.20^e^	25.71 ± 0.24^f^	0.000	0.000	0.982	0.000	0.997
∑*n* − 6*PUFA*^*c*^	4.29 ± 0.09^a^	7.23 ± 0.01^b^	11.16 ± 0.26^c^	17.65 ± 0.14^d^	24.59 ± 0.20^e^	33.96 ± 0.75^f^	0.000	0.000	0.982	0.000	0.994
18:3n-3	0.40 ± 0.14^a^	0.71 ± 0.02^b^	0.88 ± 0.07^bc^	1.07 ± 0.07^cd^	1.29 ± 0.01^d^	1.58 ± 0.06^e^	0.000	0.000	0.898	0.000	0.894
20:5n-3	3.83 ± 0.04^a^	5.02 ± 0.06^b^	5.61 ± 0.05^c^	7.11 ± 0.05^d^	9.44 ± 0.06^e^	10.24 ± 0.03^f^	0.000	0.000	0.986	0.000	0.985
22:5n-3	0.44 ± 0.05^a^	0.78 ± 0.01^b^	0.93 ± 0.06^bc^	1.10 ± 0.02^c^	0.54 ± 0.07^a^	1.60 ± 0.00^d^	0.000	0.006	0.351	0.023	0.316
22:6n-3	3.36 ± 0.09^a^	6.08 ± 0.17^b^	7.32 ± 0.08^c^	10.60 ± 0.00^d^	14.02 ± 0.24^e^	17.32 ± 0.07^f^	0.000	0.000	0.992	0.000	0.992
∑*n* − 3*PUFA*^*d*^	8.03 ± 0.29^a^	12.60 ± 0.17^b^	14.74 ± 0.21^c^	19.88 ± 0.14^d^	24.28 ± 0.22^e^	30.74 ± 0.12^f^	0.000	0.000	0.991	0.000	0.991
∑*n* − 3*LC* − *PUFA*^*e*^	7.63 ± 0.16^a^	11.88 ± 0.15^b^	13.86 ± 0.15^c^	18.81 ± 0.08^d^	23.99 ± 0.23^e^	29.15 ± 0.06^f^	0.000	0.000	0.992	0.000	0.993
ARA/EPA^f^	0.38 ± 0.01^a^	0.59 ± 0.01^b^	1.12 ± 0.03^c^	1.72 ± 0.02^d^	1.92 ± 0.02^e^	2.51 ± 0.02^f^	0.000	0.000	0.975	0.000	0.978
ARA/DHA ^g^	0.43 ± 0.01^a^	0.48 ± 0.01^a^	0.86 ± 0.02^b^	1.15 ± 0.00^c^	1.29 ± 0.02^d^	1.48 ± 0.02^e^	0.000	0.000	0.952	0.000	0.969

Data are presented as mean ± SD (*n* = 3). Values in the same row with different superscripts are significantly different (*p* < 0.05). Some fatty acids, of which the contents are minor, trace amount or not detected, such as 12 : 0, 20 : 0, 22 : 0, 24 : 0, 22:1n-9, 22:1n-11, 20:2n-6, 20:3n-6, 22:4n-6, 20:5n-6, and 20:3n-3, were not listed in this table. ^a^SFA: saturated fatty acids; ^b^MUFA: monounsaturated fatty acids; ^c^n-6 PUFA: n-6 polyunsaturated fatty acids; ^d^n-3 PUFA: n-3 polyunsaturated fatty acids; ^e^n-3 LC-PUFA: n-3 long chain poly-unsaturated fatty acid; ^f^ARA/EPA, the ratio of ARA to EPA; and ^g^ARA/DHA, the ratio of ARA to DHA.

**Table 7:** Muscle fatty acid composition of juvenile mud crab *Scylla paramamosain* fed with different dietary ARA levels (mg g^−1^ dry matter).

Fatty acids	Dietary ARA levels (g kg^−1^)						ANOVA	Linear		Quadratic	
	0.40	2.50	4.60	8.90	12.50	15.70	*p* value	*p* value	*R* ^2^	*p* value	*R* ^2^
14 : 0	0.11 ± 0.01	0.11 ± 0.01	0.09 ± 0.01	0.09 ± 0.00	0.08 ± 0.00	0.10 ± 0.01	0.135	0.097	0.111	0.044	0.252
16 : 0	2.49 ± 0.04^b^	2.92 ± 0.02^d^	2.68 ± 0.05^c^	1.93 ± 0.03^a^	2.00 ± 0.01^a^	1.96 ± 0.00^a^	0.000	0.000	0.637	0.000	0.615
18 : 0	1.35 ± 0.04^b^	1.18 ± 0.01^a^	1.26 ± 0.01^ab^	1.36 ± 0.01^b^	1.38 ± 0.06^b^	1.33 ± 0.01^b^	0.003	0.103	0.105	0.272	0.047
∑*SFA*^*a*^	3.95 ± 0.01^b^	4.21 ± 0.02^c^	4.03 ± 0.05^b^	3.38 ± 0.03^a^	3.47 ± 0.05^a^	3.38 ± 0.02^a^	0.000	0.000	0.711	0.000	0.698
16:1n-7	0.30 ± 0.02^d^	0.26 ± 0.00^cd^	0.22 ± 0.00^bc^	0.19 ± 0.01^ab^	0.16 ± 0.01^ab^	0.16 ± 0.01^a^	0.000	0.000	0.808	0.000	0.881
18:1n-9	1.75 ± 0.00^c^	1.76 ± 0.00^c^	1.62 ± 0.00^b^	1.53 ± 0.03^a^	1.52 ± 0.04^a^	1.62 ± 0.00^b^	0.000	0.001	0.449	0.000	0.769
∑*MUFA*^*b*^	2.05 ± 0.02^c^	2.02 ± 0.00^c^	1.85 ± 0.00^b^	1.72 ± 0.04^a^	1.68 ± 0.03^a^	1.79 ± 0.01^ab^	0.000	0.000	0.632	0.000	0.879
18:2n-6	0.42 ± 0.03	0.40 ± 0.01	0.39 ± 0.01	0.40 ± 0.03	0.40 ± 0.03	0.34 ± 0.01	0.214	0.047	0.176	0.107	0.159
20:4n-6	0.41 ± 0.01^a^	0.78 ± 0.01^b^	1.02 ± 0.00^c^	1.11 ± 0.04^c^	1.44 ± 0.07^d^	1.88 ± 0.02^e^	0.000	0.000	0.929	0.000	0.925
∑*n* − 6*PUFA*^*c*^	0.83 ± 0.03^a^	1.18 ± 0.01^b^	1.41 ± 0.01^c^	1.51 ± 0.06^c^	1.84 ± 0.09^d^	2.23 ± 0.03^e^	0.000	0.000	0.927	0.000	0.922
18:3n-3	0.06 ± 0.00^a^	0.08 ± 0.00^b^	0.06 ± 0.00^a^	0.05 ± 0.00^a^	0.05 ± 0.01^a^	0.05 ± 0.01^a^	0.000	0.002	0.411	0.011	0.378
20:5n-3	2.21 ± 0.01^e^	2.03 ± 0.01^c^	1.92 ± 0.01^b^	2.08 ± 0.03^cd^	2.15 ± 0.01^de^	1.53 ± 0.00^a^	0.000	0.010	0.311	0.008	0.401
22:5n-3	0.12 ± 0.01^c^	0.12 ± 0.01^bc^	0.10 ± 0.00^abc^	0.09 ± 0.00^ab^	0.10 ± 0.00^abc^	0.08 ± 0.01^a^	0.001	0.000	0.605	0.001	0.588
22:6n-3	1.79 ± 0.04^c^	1.62 ± 0.01^b^	1.50 ± 0.01^a^	1.52 ± 0.03^ab^	1.62 ± 0.01^b^	1.55 ± 0.03^ab^	0.000	0.050	0.170	0.002	0.506
∑*n* − 3*PUFA*^*d*^	4.18 ± 0.05^e^	3.85 ± 0.03^cd^	3.58 ± 0.02^b^	3.75 ± 0.01^c^	3.92 ± 0.02^d^	3.22 ± 0.03^a^	0.000	0.002	0.415	0.011	0.378
∑*n* − 3*LC* − *PUFA*^*e*^	4.12 ± 0.05^e^	3.77 ± 0.03^cd^	3.52 ± 0.01^b^	3.70 ± 0.01^c^	3.87 ± 0.01^d^	3.17 ± 0.03^a^	0.000	0.004	0.384	0.016	0.346
ARA/EPA^f^	0.19 ± 0.00^a^	0.38 ± 0.00^b^	0.54 ± 0.00^c^	0.53 ± 0.03^c^	0.67 ± 0.03^d^	1.23 ± 0.01^e^	0.000	0.000	0.815	0.000	0.852
ARA/DHA^g^	0.23 ± 0.01^a^	0.48 ± 0.01^b^	0.68 ± 0.01^c^	0.73 ± 0.03^c^	0.89 ± 0.05^d^	1.21 ± 0.03^e^	0.000	0.000	0.907	0.000	0.902

Data are presented as mean ± SD (*n* = 3). Values in the same row with different superscripts are significantly different (*p* < 0.05). Some fatty acids, of which the contents are minor, trace amount or not detected, such as 12 : 0, 20 : 0, 22 : 0, 24 : 0, 22:1n-9, 22:1n-11, 20:2n-6, 20:3n-6, 22:4n-6, 20:5n-6, and 20:3n-3, were not listed in this table. ^a^SFA: saturated fatty acids; ^b^MUFA: monounsaturated fatty acids; ^c^n-6 PUFA: n-6 polyunsaturated fatty acids; ^d^n-3 PUFA: n-3 polyunsaturated fatty acids; ^e^n-3 LC-PUFA: n-3 long chain poly-unsaturated fatty acid; ^f^ARA/EPA, the ratio of ARA to EPA; and ^g^ARA/DHA, the ratio of ARA to DHA.

## Data Availability

Data were available from the corresponding author by reasonable request.

## Supplementary Materials

Supplementary Materials Supplementary file for this manuscript: real-time quantitative PCR primers for lipid-metabolism genes of mud crab are attached.
