# Supplementary material for: Alteration of Growth Performance, Antioxidant Capacity, Tissue Fatty Acid Profiles, and Lipid Metabolism of Mud Crab (Scylla paramamosain) Juvenile in Response to Different Dietary Arachidonic Acid Levels
Source: Aquac Nutr. 2022 Nov 23;2022:6038613. doi: 10.1155/2022/6038613 (PMC10281821; doi:10.1155/2022/6038613)
Supplement: Supplementary Materials — Supplementary file for this manuscript: real-time quantitative PCR primers for lipid-metabolism genes of mud crab are attached. [file 6038613.f1.docx]

**Alteration of** **growth performance,** **antioxidant capacity, tissue fatty acid profiles and lipid metabolism of mud crab (*Scylla paramamosain*)** **juvenile in response to different dietary arachidonic acid levels**

Fang Fang ^1,2,a^, Ye Yuan ^3,a^, Min Jin ^1,2^, Yingying Zhang ^1,2^, Tingting Zhu ^1,2^, Jiaxiang Luo ^1,2^, Zheng Yang ^1,2^, Chen Guo ^1,2^, Lefei Jiao ^1,2^, Xiaojun Yan^1,2^, Qicun Zhou ^1,2*^

*^1^ Laboratory of Fish and Shellfish Nutrition, School of Marine Sciences, Ningbo University, Ningbo 315211, China*

*^2^ Key Laboratory of Aquaculture Biotechnology Ministry of Education, Ningbo University, Ningbo 315211, China*

*^3^ Guangdong Provincial Key Laboratory of Marine Biotechnology, Institute of Marine Sciences, Shantou University, Shantou 515063, China*

***Corresponding author:**

*E-mail address*: zhouqicun@nbu.edu.cn (Qicun Zhou)

^a^ These authors contributed equally to this work as first author.

SUPPLEMENTARY TABLE 1: Real-time quantitative PCR primers for lipid-metabolism genes of mud crab

| Gene | Nucleotide sequence (from 5′ to 3′) | Size (bp) | GenBank no. |
| --- | --- | --- | --- |
| *fas* | F: CATTCTGGGCATCAAGGA | 263 | HM217807.1 |
|  | R: TGTTGGAGTCAGGGAGCA |  |  |
| *acc* | F: AACTGTACGGTGGAGTGTGT | 238 | PRJNA634782 |
|  | R: TCCTGTCCATCTGCAACCTT |  |  |
| *6gpd* | F: TCAATCGCACCACGGAGA | 124 | PRJNA634782 |
|  | R: AGCATAACACGCCTCGGT |  |  |
| *g6pd* | F: AATGAGAGGAAGGCTGAGGT | 194 | PRJNA634782 |
|  | R: CGGTCGCCATAAGTCAAGTC |  |  |
| *fabp1* | F: CACGCCATCCAACTCAAC | 109 | JQ824129.1 |
|  | R: CATCACCATTCCCACACC |  |  |
| *fabp4* | F: ATTGGTGAGATTTGCCGCTA | 218 | PRJNA634782 |
|  | R: ACAAAGCCGACTGCTCCTAC |  |  |
| *srb2* | F: CTGCTCGCTTTATCACACCC | 153 | LT797149.2 |
|  | R: GAACCACCATTCCGCTTGTT |  |  |
| *hsl* | F: TTTGTCAGTGGCAGTGTTGG | 151 | PRJNA634782 |
|  | R: CCTCAGTCTGTCCATGGTGT |  |  |
| *aco1* | F: TGAGGACAGATACGCTAACG | 156 | PRJNA634782 |
|  | R: TCTGTGCCAGTGGGTGAGGT |  |  |
| *aco3* | F: TTGATGATGTGGGCTGCTTT | 132 | PRJNA634782 |
|  | R: TCTGTGCCAGTGGGTGAGGT |  |  |
| *cpt1* | F: CTTGTCCGTCGCCTTGTAGA | 222 | PRJNA634782 |
|  | R: ATTGAGCAGCCGACCCTTAT |  |  |
| *cpt2* | F: GCACAACTCCCAAGTCATTC | 164 | PRJNA634782 |
|  | R: AAAATGTCTGGCATAGGGGA |  |  |
| *srebp1* | F: AGTGACACGGCAACTCCTG | 113 | MH910503.1 |
|  | R: ACAAACATTCCCTCGTACATC |  |  |
| *hnf4α* | F: GCACCTCTCCAGACAGTTCT | 250 | PRJNA634782 |
|  | R: CAGAATCATGTCCTGCAGCC |  |  |
| *β-actin* | F: GCGGCAGTGGTCATCTCCT | 168 | KC795683.1 |
|  | R: GCCCTTCCTCACGCTATCCT |  |  |

*fas*, fatty acid synthase; *acc*, acetyl-CoA carboxylase; *6pgd*, 6-phosphogluconate dehydrogenase; *g6pd*, glucose-6-phosphate dehydrogenase; *fabp*, fatty acid binding protein; *srb2*, scavenger receptor class 2; *hsl*, hormone-sensitive lipase; *aco*, 1-aminocyclop ropane-1-carboxylic acid oxidase; *cpt*, carnitine palmitoyltransferase; *srebp1*, sterol regulatory element-binding protein 1; *hnf4α*, hepatocyte nuclear factor 4α.
